# A Prospective Study Examining the Consequences of Family Rejection for Sexual and Gender Minority Youth

**DOI:** 10.1111/fare.70286

**Published:** 2026-07-26

**Authors:** Weiman Xu, Lorey A. Wheeler, Heather Littleton, Seungju Kim, Rayni Thomas, Joseph Gardella, Jillian R. Scheer, Caitlin Ryan, Alexis Chavez, Antonia Barba, Caroline Spitz, Katie M. Edwards

**Affiliations:** 1 University of Nebraska-Lincoln, Lincoln, Nebraska, USA; 2 University of Colorado, Colorado Springs, Colorado Springs, Colorado, USA; 3 University of Illinois Urbana-Champaign, Champaign, Illinois, USA; 4 University of Michigan, Ann Arbor, Michigan, USA; 5 University of Rhode Island, Kingston, Rhode Island, USA; 6 Family Acceptance Project and San Francisco State University, San Francisco, California, USA; 7 Inform Transform, New York, New York, USA

**Keywords:** family rejection, family-oriented care, LGBTQ, mental health, sexual and gender minority youth

## Abstract

**Objective:**

This study examined the prospective associations between family rejection and several resilience factors and deleterious outcomes among a racial and gender diverse sample of sexual and gender minority youth (SGMY).

**Background:**

Family remains a primary developmental context during adolescence, and there is an urgent need to better understand how familial responses to SGMY identities influence resilience factors and deleterious outcomes.

**Method:**

Participants were 285 SGMY aged 15 to 18 (54.4% youth of color, 57.5% trans and gender diverse) from across the United States who completed a baseline survey and a follow-up survey about 4 months later as part of a larger study examining an online prevention program for teen dating violence (TDV) and alcohol use for SGMY.

**Results:**

From baseline to 4 months later, higher family rejection was prospectively related to increased internalized homo/bi/transphobia, TDV perpetration and victimization, and depression. Further, higher family rejection was prospectively related to decreased hope for the future and SGM sense of community.

**Conclusion and Implications:**

These findings underscore the critical need for family-oriented programs to reduce family rejecting behaviors and increase family accepting and supportive behaviors among caregivers of SGMY.

## 1 | Introduction

Sexual and gender minority youth (SGMY) including SGM adolescents, experience epidemic rates of behavioral (e.g., alcohol use), emotional (e.g., depression, suicidality), and relational (e.g., dating violence victimization and perpetration) problems (Ancheta et al. 2021; Ash-Houchen and Lo 2018; Centers for Disease Control and Prevention [CDC] 2023; Mustanski 2015). These problems, in part, have been attributed to social (minority) stress experienced by SGMY populations due to their stigmatized status related to their sexual orientation, gender identity, and gender expression (SOGIE; Edwards et al. 2020; Meyer 2003; Testa et al. 2015). A growing body of research underscores the pivotal role of the family environment in either mitigating or exacerbating these risks. In particular, experiences of negative responses to identity disclosure in the family context can serve as a potent source of minority stress, with enduring consequences for mental and relational health (Palmer and Francis 2024; Pollitt et al. 2023). Given that the family remains a primary developmental context during adolescence, there is an urgent need to better understand how familial responses to SGMY identities influence resilience factors (e.g., hope for the future) and deleterious outcomes (e.g., dating violence).

Family rejection, defined as caregivers' or other family members' attitudes and behaviors that demonstrate disapproval of and/or efforts to change, prevent, minimize or deny the child's SOGIE, has been identified as a significant interpersonal minority stressor within the family context (Ryan 2019a). Family rejection is characterized by a withdrawal of warmth and love and/or engagement in hostility and aggression based on youth's SOGIE. Family rejection may exacerbate experiences of societal oppression; thus, contributing to deleterious outcomes, such as violence, depressive symptoms, internalized homo/bi/transphobia, and risk behavior, while also potentially diminishing access to resilience factors like hope for the future and community connectedness (Meyer 2003). Despite the importance of the family context for SGMY, there is a paucity of research that has identified what might be the most salient risk and resilience factors that could be targeted in preventive-intervention efforts supporting positive development among SGMY experiencing rejection.

### 1.1 | Sociodemographic Variability in Family Rejection

Research suggests that the majority (50% to 70%+) of SGMY experience some level of family rejection (Richter et al. 2017; Salerno et al. 2023; VanBergen and Love 2022), with experiences being more common among trans and gender minority youth compared to cisgender sexual minority youth, and among SGMY of color compared to white, non-Latine SGMY (Hidalgo et al. 2019; Testa et al. 2015; Toomey et al. 2017). Research also suggests that family reactions to their SGMY's identity can vary widely from completely rejecting to completely accepting, with most families being ambivalent or in the process of learning about their child's SOGIE (Ryan et al. 2009; Ryan et al. 2010). These disparities of family rejection may reflect intersecting forms of marginalization and variation in cultural or familial beliefs about gender and sexuality (Ryan et al. 2010).

However, much of the existing research has focused on a limited range of identities, often examining racial-ethnic identity or gender identity in isolation (Sulla et al. 2025). Less attention has been given to the broader sociodemographic variability in family rejection experiences among SGMY. There is a need for more intersectional and comprehensive subgroup comparisons to identify youth most at risk for family rejection and to inform culturally responsive prevention and intervention strategies. To address this gap, our first research question examined the prevalence of family rejection among SGMY and whether its prevalence varies across key sociodemographic subgroups, including age, racial-ethnic identity, gender identity, and sexual orientation.

### 1.2 | Family Rejection and Youth Outcomes

The present study is guided by an integrated theoretical framework that draws on parental acceptance-rejection theory (PAR theory; Rohner 2004), marginalized family identity theory (Farr et al. 2025), and a life course perspective for LGBTQ+ individuals (Meslay et al. 2025) to understand the short-term impacts of family rejection on SGMY's well-being. At the intrapersonal level, PAR theory posits that perceived rejection from parents through emotional coldness, hostility, or disapproval undermines youth's psychological adjustment. Applied to the experiences of SGMY, PAR theory provides a compelling framework for understanding the distinct and amplified harms of family rejection that is explicitly rooted in a youth's SOGIE status. Adolescence is a period characterized by identity exploration, emotional sensitivity, and continued dependence on family (Arnett 2000; Steinberg 2014). Family rejection may represent a failure to cultivate an affirming shared family identity that includes and validates youth's sexual and gender identities (Farr et al. 2025).

At the relational level, marginalized family identity theory (Farr et al. 2025) emphasizes the identity-based dynamics within the family system, offering a complementary lens for understanding the unique challenges SGMY face during SOGIE disclosure. Because family rejection targets youths' emerging sense of self, it can be especially damaging during identity formation. In particular, identity-based rejection undermines core developmental needs for affirmation and belonging. Marginalized family processes, such as lack of affirming discourse (e.g., avoiding conversations about a youth's SOGIE), boundary ambiguity (e.g., uncertainty about whether and how a youth's identity can be expressed within family roles or relationships), and stigmatization (e.g., framing SGM identities as shameful), can impede youth identity development and contribute to maladaptive outcomes. Family rejection during this developmental period can have especially detrimental consequences for SGMY who have disclosed their SGOIE status to at least one family member, as support may be conditional on suppressing or denying one's authentic identity (Russell and Fish 2016). In line with PAR theory, such rejection disrupts the satisfaction of basic emotional needs and contributes to heightened psychological vulnerability, thereby increasing the risk for a range of adverse outcomes for SGMY, including depression, suicidality, substance use, and relational difficulties.

From the life course perspective for LGBTQ+ individuals (Meslay et al. 2025), adolescence is a critical turning point for identity disclosure. According to Meslay et al. (2025), SOGIE disclosure marks a significant departure from cis-hetero-normative expectations and redefines one's relationships within the family system. Because this moment often challenges established relational norms, youth are particularly vulnerable to rejection during this time, making the family's response highly consequential. The life course principle of *linked lives* further suggests that such turning points are not experienced in isolation but are deeply embedded in familial and generational relationships, shaping identity development and future relational connections (Meslay et al. 2025). These theoretical perspectives underscore that family rejection is both a psychological and relational stressor that may relate to short-term changes in both deleterious and resilience-promoting outcomes for SGMY.

Supporting these theory-based assertions, previous qualitative and quantitative research finds that family rejection among SGMY is related to TDV perpetration and/or victimization (Ronzón-Tirado et al. 2022; Sterzing et al. 2017), sexual risk taking (Bird et al. 2017; Ryan et al. 2009), alcohol and drug use (Gamarel et al. 2020; Klein and Golub 2016; Ryan et al. 2009; Yang et al. 2019), mental health problems (Abreu, Lefevor, et al. 2023; Klein and Golub 2016; Meanley et al. 2021; Munroe 2018; Ryan et al. 2009), and suicidality (de Lange et al. 2022; VanBergen and Love 2022). Indeed, in a study of SGMY with clinically significant depressive and suicidal symptoms, Diamond et al. (2011) found that family rejection was among the most commonly cited sources of psychological distress, and many SGMY expressed a desire for greater parental acceptance. However, nearly all this research has been cross-sectional.

Only a handful of published studies have examined the prospective association between family rejection and SGMY's deleterious outcomes. Pachankis et al. (2018) found in a sample of young sexual minority men that “unfinished business with fathers” prospectively related to next-year depression symptoms but was not prospectively related to social anxiety or alcohol abuse. In a study with SGMY surveyed across a 5.5-year period, researchers found that youth with low and no family support reported more mental health distress at all time points compared to youth with high support (McConnell et al. 2016). Finally, in a study of SGM individuals ages 16 and over in the United Kingdom, researchers found that family support predicted fewer sleep problems at the two-year follow-u p (Chum et al. 2021). Beyond contributing to deleterious outcomes, family rejection may also erode access to resilience-promoting resources that are uniquely salient for SGMY. For example, family rejection may limit a youth's ability to safely disclose their sexual or gender identity to others outside of the family or to extended family (outness), diminish their optimism about the future (hope), and reduce their sense of belonging to the broader LGBTQ+ community. When caregivers react with hostility or withdrawal, youth may become reluctant to be open about their identities even in otherwise safe contexts, reinforcing internalized stigma and isolation (Russell and Fish 2016). Similarly, lack of emotional support from family can undermine youth's capacity to envision a fulfilling future as an LGBTQ+ person, especially during a developmental stage when future orientation is taking shape (Moe et al. 2023). Finally, youth who experience family rejection may feel alienated not only within their families but also from affirming peer networks, particularly if rejection conveys that LGBTQ+ identity is shameful or unacceptable (Frost and Meyer 2012; McConnell et al. 2015). Thus, family rejection not only contributes to risk but may actively block access to critical sources of intrapersonal and interpersonal resilience during adolescence.

Given high rates of adjustment problems for the SGMY population (e.g., Ancheta et al. 2021; CDC 2023), additional research is needed to develop a better understanding of the prospective effects of family rejection. To date, most empirical studies on family rejection and youth outcomes have focused narrowly on youth mental health outcomes, with less attention paid to other critical domains of youth well-being. Yet theoretical frameworks (e.g., PAR theory, marginalized family identity theory, and life course perspective) and emerging evidence suggest that family rejection may be related to a broader constellation of outcomes among SGMY, including dating violence, risk behaviors, psychological/emotional problems, and LGBTQ+ specific risk and resilience outcomes. Moreover, despite growing recognition of these multifaceted consequences, few studies have adopted a prospective design to examine how family rejection shapes both deleterious and resilience-promoting outcomes over time, which limits our ability to understand longitudinal influences of family rejection on SGMY well-being, and develop evidence-based interventions. To address these gaps, our second research question examined the prospective associations between family rejection and changes in both risk and resilience indicators among a diverse sample of SGMY.

### 1.3 | Current Study

The lack of prospective research limits our understanding of the consequences of family rejection on varying domains of deleterious outcomes among SGMY. Moreover, to our knowledge, no research has focused on the potential detrimental effects of family rejection on SGM-specific risk (internalized homo/bi/transphobia) or resilience factors (e.g., hope for the future as an SGM person and LGBTQ+ sense of community) that may be critical in reducing risk for negative outcomes. We addressed these gaps in the current study. Guided by existing theoretical frameworks, the present study uses a prospective design to empirically examine short-term associations between family rejection and multiple domains of youth adjustment. More specifically, building off extant literature, this study used a diverse sample of SGMY, comprehensive set of salient adjustment domains for SGMY, and prospective methodology to answer two research questions:

**RQ1**. *How common is family rejection among SGMY and does it vary among sociodemographic subpopulations (i.e., age, race and ethnicity, gender identity, and sexual orientation)?*

**RQ2**. *How does family rejection prospectively relate to various domains of adjustment and well-being among SGMY: (a) TDV perpetration and victimization; (b) risk behavior (i.e., alcohol use, drinking to cope, sexual risk behaviors); (c) psychological/emotional problems (depressive symptoms, impulse control difficulties), and (d) LGBTQ*+ *risk/resilience factors (i.e., internalized homo/bi/transphobia, outness, hope for the future, sense of LGBTQ*+ *community)?*

In addressing RQ2, we controlled for the experimental design of the larger study (experimental condition), baseline measurements of the dependent variables (DV), as well as examined theoretical covariates (Sulla et al. 2025) of the association between family rejection and the DVs (i.e., youth age, economic hardship, race/ethnicity, gender identity, and sexual orientation).

## 2 | Method

### 2.1 | Participants

Data came from a larger randomized controlled trial evaluating the efficacy of an online live-facilitated nine-session group program for SGMY who were currently dating. In the larger study, participants were 304 SGMY in the United States, ranging in age from 15 to 18 years old randomized to either control or treatment (Promoting Resilient Youth with Strong Hearts and Minds [PRYSHM]; see Edwards et al. 2025 for more details). For the current study, we selected the subsample of youth who were out to at least one family member (*n* = 285 SGMY, *M*_age_ = 16.53, SD_age_ = 0.88), and thus the family rejection questions were applicable to them. Among the 285 SGMY, 155 (54.4%) identified as a youth of color, 164 (57.5%) as transgender and gender diverse, and 75 (26.3%) as monosexual. Participants were geographically diverse, residing across all four US Census regions: South (*n* = 104; 36.5%), West (*n* = 70; 24.6%), Midwest (*n* = 67; 23.5%), and Northeast (*n* = 44; 15.4%). Most participants (*n* = 253; 88.8%) lived in metropolitan areas, including those in urban cores (*n* = 238; 83.5%) and those commuting into urban areas (*n* = 15; 5.3%). Smaller proportions resided in micropolitan (*n* = 24; 8.5%), small town (*n* = 5; 1.8%), or rural areas (*n* = 3; 1.1%). Over half of youth reported at baseline (Time 1; T1) having one dating partner (*n* = 164, 57.5%), with the most common relationship status being in a serious/committed dating relationship (*n* = 137, 48.1%) (see Table 1 for sample demographics).

### 2.2 | Procedures

All study procedures were approved by the University of Nebraska Lincoln Institutional Review Board. Participants were recruited from across the United States via ads posted on social media (largely Instagram boosted posts), an online dating site, and from LGBTQ+ organizations identified from the CenterLink directory (https://lgbtqcenters.org) who agreed to post information about the study. Interested individuals were directed to a screener survey. The screener assessed eligibility criteria which included: (1) identify as an SGMY; (2) be between 15 and 18 years of age (and not enrolled as a college student); (3) read and speak English; (4) reside in the United States; (5) consistent access to a device with high speed Internet access; (5) ability to attend the nine, 60-min online teleconference sessions; (6) in a dating relationship during the past 3 months; (7) have safe means to communicate via email and/or text; (8) not be at high risk for suicide; and (9) be available to participate in the program during one of the times the program was offered. A multi-stage gated procedure was implemented to ensure that fraudulent responders were identified and not enrolled. Specifically, screener responses were evaluated for indicators of fraudulent response patterns (e.g., report of a residential address that does not exist; IP address does not match location) and those representing clearly fraudulent response patterns were not contacted further. Next, a Zoom screen procedure was utilized where participants were asked to verify and spell their name, address, and phone number, turn on their camera and show their unobscured face, and answer specific questions (e.g., “What does it mean to you to be LGBTQ+?”). Any individual whose responses were determined to be fraudulent by two raters were not invited to participate further.

A waiver of parental consent was obtained due to the potential for risk of harm associated with participation leading to parental knowledge of participants' SGM status. Assent to participate was obtained from all participants. Participants were compensated $10 for the baseline survey (T1) and $30 for the 3-month post intervention follow-u p survey administered approximately 5.5 months later (Time 3 from the original study). The intervention occurred between T1 and T3 (Time 2 was an immediate post-test, 2.5 months post-baseline). Of the participants included at baseline (T1), 82.8% were retained at the 3-month follow-up survey administered 5.5 months after baseline (T3). The intervention was focused on building resilience among SGMY and did not include family members. As such, the intervention would theoretically have no impact on family rejection. However, given this is an important aspect of the research design of the larger study, we included condition (0 = *waitlist*, 1 = *PRYSHM*) as a covariate in analyses to control for intervention effects on the dependent variables.

### 2.3 | Measures

#### 2.3.1 | Sociodemographic Background (T1)

Youth reported on a series of questions about their sociodemographic background (age, race/ethnicity, sex assigned at birth, gender identity, and sexual orientation). For analytic purposes in testing RQ1, we created categorical variables for grouping youth. For race and ethnicity, youth responded by choosing all identities that apply (American Indian or Alaska Native, Asian, Black or African American, White, Hispanic or Latino/a/e). We used those responses to code race/ethnicity into six categories: American Indian or Alaska Native, non-Latino/a/x; Asian, non-Latino/a/x; Black or African American, non-Latino/a/x; White, non-Latino/a/x; Hispanic or Latino/a/x only; and Multiracial (includes Hispanic or Latino/a/x).

To represent gender identity, we used the sex assigned at birth (i.e., female, male, intersex) and gender identity (i.e., boy/man, girl/woman, trans, trans girl/woman, trans boy/man, genderqueer, non-binary, unsure/questioning, or choose to self-identify) questions. This coded variable included four categories: (1) male adolescents who identified as cisgender (sex assigned at birth = male [AMAB]; gender identity = boy/man [cis]); (2) female adolescents who identified as cisgender (sex assigned at birth = female [AFAB]; gender identity = girl/woman [cis]); (3) male adolescents (AMAB) who identified as trans and gender diverse (TGD; i.e., chose any other gender identity categories); and (4) female adolescents (AFAB) who identified as trans and gender diverse (TGD).

The sexual orientation (heterosexual/straight, gay, lesbian, pansexual, bisexual, queer, asexual, demisexual, questioning/unsure of my sexual orientation, or other [choose to self-identify]) question was used to create a binary variable: 0 = *monosexual* and 1 = *non-monosexual*. The *monosexual* category includes heterosexual/straight, gay, and lesbian. The remaining youth were coded as non-monosexual. Our choice of terms was guided by recent work by Santos and Craig (2024), recommending the use of monosexual as a neutral term to describe and understand social structures that may contribute to oppression across groups.

#### 2.3.2 | Family Rejection (T1)

Questions from the Family Rejection Scale (Ryan et al. 2009) were used to assess SOGIE-based rejection from family (4 items; e.g., “Makes you feel like you are bad because you are an LGBTQIA2S+ person”). Items were rated on a 4-point rating scale (0 = *Never* to 3 = *Often*). A mean composite was created with higher scores indicating higher family rejection (T1 *α* = 0.84).

#### 2.3.3 | Teen Dating Violence (TDV) Perpetration and Victimization (T1, T3)

The Measure of Adolescent Relationship Harassment and Abuse (Rothman et al. 2022) was used to assess teen dating violence (TDV) perpetration (37 items; social control, physical abuse, sexual abuse, isolation, cyber control, and intimidation; e.g., “I insulted their looks, clothes, or appearance, to make them feel bad, embarrassed, or insecure.”) and victimization (34 items; privacy control, social control, physical abuse, sexual abuse, intimidation; e.g., “They spread rumors, gossip, or secrets about me using texts, social media, or apps.”) during the past 3 months. Items were rated using a slider scale of frequency (0–100). A composite score was created representing a sum across all items for TDV victimization (T1 *α* = 0.87; T3 *α* = 0.85) and perpetration (T1 *α* = 0.88; T3 *α* = 0.56).

#### 2.3.4 | Alcohol Use (AU) (T1, T3)

Two items from the Monitoring the Future Survey (Johnston et al. 2022) were adapted to assess AU frequency ranging from 0 = 0 times to 9 = 40+ times (e.g., “On how many occasions [if any] have you had alcoholic beverages to drink [more than just a few sips]in the past 3 months”). We recoded the response scale using the mid-point of each response option (e.g., 0 coded as 0; 8 = 20–39 times, coded as 29.5; 9 = 40+ coded as 40) and then calculated a mean score indicating frequency of recent alcohol use.

#### 2.3.5 | Drinking to Cope (T1, T3)

The coping subscale (five items) of the Drinking Motivations Questionnaire-Revised (DMQ-R; Cooper 1994) was used to assess use of alcohol as a coping mechanism (e.g., “You drink … to forget your worries.”) The items were rated from 0 = *Almost never or never* to 5 = *Almost always or always*. A mean composite was calculated, with high scores representing greater use of alcohol to cope (T1 *α* = 0.94; T3 *α* = 0.95).

#### 2.3.6 | Sexual Risk Behaviors (T1, T3)

Four items adapted from the Youth Risk Behavior Surveillance System (YRBS; Mpofu et al. 2023) and Sexual Risk Survey (SRS; Turchik and Garske 2009) were used to capture the frequency of risky sexual behaviors (e.g., “How many times have you or your partner used alcohol or drugs before or during sex?”). We did not use an existing measure due to concern about length (specifically the SRS) and concerns expressed by our youth advisory board that YRBS and SRS wording was not inclusive and/or was heteronormative. Participants used a sliding scale to indicate the frequency with which they have engaged in the behavior in the past 3 months, from 0 to 30 times. A composite score was created by recoding each item (0, 1, and 2 or more) and then creating a sum (T1 *α* = 0.68; T3 *α* = 0.77). Higher scores indicate greater sexual risk behavior.

#### 2.3.7 | Depressive Symptoms (T1, T3)

We used 8 items (the item assessing suicidality was omitted) from the Patient Health Questionnaire-9 adapted for Adolescents (PHQ-A; Johnson et al. 2002) to assess depressive symptomology over the past two weeks (e.g., “Feeling down, depressed, irritable, or hopeless?”) A composite score was created by summing all items, with higher scores representing more depressive symptoms (T1 *α* = 0.88; T3 *α* = 0.90).

#### 2.3.8 | Impulse Control Difficulties (T1, T3)

We used the 3-item impulse control difficulties subscale from the Difficulties in Emotion Regulation Scale (Kaufman et al. 2016) to assess impulse control difficulties (e.g., “When I am upset, I have difficulty controlling my behaviors.”) Items were rated from 1 = *Almost never (0%–10%)* to 5 = *Almost always (91%–100%)*. A mean composite score was calculated, with higher scores representing more impulse control difficulties (T1 *α* = 0.83; T3 *α* = 0.84).

#### 2.3.9 | Internalized Homo/Bi/Transphobia (T1, T3)

Four items from the LGBTQ National Teen Survey (Watson et al. 2020) were used to measure internalized homo/bi/transphobia (e.g., “Whenever I think a lot about being LGBTQIA2S+, I feel critical of myself”). Youth rated items using a 4-point Likert scale (1 = *Strongly disagree* and 4 = *Strongly agree*). A mean composite was calculated with higher scores indicating higher internalized homo/bi/transphobia (T1 *α* = 0.73; T3 *α* = 0.75).

#### 2.3.10 | Outness (T1, T3)

The Outness Inventory (Mohr and Fassinger 2000) is a measure that captures the degree to which LGBTQ individuals have revealed to different people in their life their sexual orientation and gender identity, respectively (11 items for each domain). The original inventory is comprised of three subscales: out to family (four items), out to world (five items), and out to religion (two items). We added 8 items to the original measure to include other relationships (e.g., classmates at school, non-LGBTQIA2S+ friends, doctors and other healthcare providers) based on input from our youth advisory board and core project team who felt that the list should be more inclusive. Also, in the out to the world subscale, three out of the five items were asking about outness to other family members beyond caregivers. Thus, in the current study, we created two composite scores by computing the proportion of outness to all family members (seven items; including outness to mother, stepmother, father, stepfather, siblings/stepsiblings/half-siblings, cousins, and grandparents or other extended family) and the remaining items (i.e., outness to the world; 12 items; e.g., classmates at school), respectively. For participants when these items are not applicable, they were coded as missing in the dataset. Higher scores indicate higher levels of outness regarding one's sexual orientation and gender identity, respectively. Of note, the gender identity items were only administered to participants who indicated that they were TGD.

#### 2.3.11 | Hope for the Future as an LGBTQ Person (T1, T3)

Four questions from the Human Rights Campaign (HRC) Growing Up LGBT in America Youth Survey Report (Watson et al. 2020) were used to assess youth hope for the future as an LGBTQ+ person (e.g., “Are you able to see yourself in the future as a happy or successful LGBTQIA2S+ adult?”). Items were rated on a 4-point rating scale (1 = *Definitely no*; 2 = *Somewhat no*; 3 = *Somewhat yes*; and 4 = *Definitely yes*). A mean composite was calculated, with higher scores indicating higher hope and optimism (T1 *α* = 0.61; T3 *α* = 0.68).

#### 2.3.12 | Sense of LGBTQ+ Community (T1, T3)

Two subscales from the Psychological Sense of LGBTQ Community Scale (Lin and Israel 2012) membership (3 items) and needs fulfillment (3 items), were used to assess LGBTQ+ sense of community (e.g., “How much do you feel that you are a member of the LGBTQ community?”). Response options ranged from 1 = *none* to 4 = *a great deal*. Given the moderate to strong correlations between the two subscales in our sample (*r* = 0.55, *p* < 0.001 at T1; *r* = 0.64, *p* < 0.001 at T3), and in line with the original authors' approach of computing an overall score, a mean composite was calculated of these two subscales, with higher scores indicating higher community connection (T1 *α* = 0.86; T3 *α* = 0.89).

### 2.4 | Data Analysis Plan

To answer RQ1 about levels and variation in family rejection, we conducted ANOVA and *t*-tests using SPSS 29.0. To answer RQ2, we used path analysis in *Mplus* 8.8 (Muthén and Muthén 1998–2017). The independent variable (IV) was family rejection measured at baseline (T1). We grouped together the dependent variables (DV) measured at T3 (as defined in the current study as 5.5-months post baseline) by construct to reduce the number of models estimated. Specifically, Model 1 includes TDV perpetration and victimization; Model 2 includes risk behavior (i.e., alcohol use, drinking to cope, and sexual risk behavior); Model 3 includes psychological/emotional problems (i.e., depressive symptoms and impulse control difficulties); and Model 4 includes LGBTQ+ risk/resilience factors (i.e., internalized homo/bi/transphobia, sexual orientation outness, gender identity outness, hope for the future, and sense of LGBTQ+ community). Grouping DVs by construct helped reduce experiment-wise error while maintaining theoretical coherence. A single omnibus model was not feasible due to (a) sample size constraints, and (b) variation in outcome distributions (e.g., count vs. continuous), which required different estimators in analyses. Potential theoretical covariates were examined as potential third variable explanations of findings, including youth age (continuous), economic hardship (continuous), race (white [not Latine] vs. youth of color), gender identity (cisgender vs. trans/gender diverse), and sexual orientation (monosexual vs. non-monosexual). Guided by recommendations from Spector and Brannick (2011), we tested models with and without the full set of covariates to determine whether covariates ruled out possible third variable explanations of findings. Patterns of findings did not vary between the models, thus final models presented do not include these covariates. T1 measures of DVs and intervention condition (0 = *waitlist*, 1 = *PRYSHM*) were always included in the model as covariates. Missing data were handled using full information maximum likelihood (FIML) estimation in *Mplus*, which allows for the inclusion of all available data under the assumption of missing at random. Descriptive statistics and correlations for all study variables are presented in Table 2. As indicators of effect size, we used the incidence rate ratio (IRR; closer to 1 represents a smaller effect) for categorical variables and *R*^2^ for explained variance in models (small = 0.02, medium = 0.13, large = 0.26; Cohen 1992).

## 3 | Results

### 3.1 | RQ1: Variation of Family Rejection Across Demographic Groups

Participants reported a family rejection mean score of 1.13 (SD = 0.90) on a scale of 0 (*never*) to 3 (*often*). Findings indicated significant differences in family rejection experienced by SGMY across different gender identity groups (Table 3). Specifically, AMAB cis reported lower levels of family rejection than TGD youth with male sex assigned at birth (*M*_*AMAB cis*_ = 0.80, *M*_*AMAB TGD*_ = 1.60, *p* = 0.042). There were no significant differences in family rejection by age, race and ethnicity, or sexual orientation.

### 3.2 | RQ2: Family Rejection and SGMY Outcomes

Starting with TDV perpetration and victimization, there was adequate model fit (Model 1; Table 4). Path analysis findings indicated a positive association between family rejection and changes in TDV perpetration and victimization 5.5 months later. Specifically, for TDV perpetration, the incidence rate ratio (IRR = 1.34) indicated that for each one-unit increase in family rejection, the expected count increased by 34%. For TDV victimization, the IRR of 1.42 indicated that for each one-u nit increase in family rejection, the expected count increased by 42%.

Turning to risk behavior, there was adequate model fit (Model 2; Table 4). For alcohol use, drinking to cope, and sexual risk behavior, no significant findings emerged. Our findings did not provide evidence of links between family rejection and risk behavior for SGMY.

Turning to psychological/emotional problems (depressive symptoms and impulse control difficulties), there was adequate model fit (Model 3, Table 4). Findings suggested that high levels of family rejection related to increased depressive symptoms 5.5 months later, explaining a significant proportion of variance (21%). Our findings did not provide evidence of links with impulse control difficulties.

For LGBTQ+ risk and resilience factors, there was adequate model fit (Model 4, Table 4). Findings suggested that higher levels of family rejection related to increased internalized homo/bi/transphobia 5.5 months later, explaining a significant proportion of variance (37%). Higher levels of family rejection related to decreased hope for the future as an LGBTQ+ person, explaining a significant proportion of variance (31%). Higher levels of family rejection also related to decreased sense of LGBTQ+ community 5.5 months later, explaining a significant proportion of variance (37%). Our findings did not provide evidence of links with outness.

## 4 | Discussion

The purpose of the current study was to document variation in family rejection and examine how family rejection prospectively related to changes in various outcomes 5.5 months later in a diverse sample of SGMY. The prospective design is a strength of the current study given that it allows us to make stronger assertions regarding the shorter-term consequences of family rejection on SGMY outcomes. To our knowledge, this is only one of a handful of studies to have examined the prospective associations of family rejection on a myriad of outcomes over time among SGMY. While the current study did not seek to develop new theoretical models, its findings offer empirical support for key propositions from existing frameworks and highlight opportunities for future conceptual refinement, particularly regarding shorter-term pathways linking family rejection to resilience and promoting well-being as well as deleterious outcomes among diverse SGMY.

### 4.1 | Variation in Levels of Family Rejection

Findings revealed that family rejection varied significantly by gender identity, with trans and gender diverse (TGD) youth with male sex assigned at birth reporting higher levels of family rejection than their cisgender peers with male sex assigned at birth. This finding aligns with prior research indicating TGD youth are at heightened risk for rejection, discrimination, and victimization within their families and broader social contexts (James et al. 2016; Olson et al. 2016; Simons et al. 2013). A key explanation for this disparity lies in the concept of multiple marginalization, wherein TGD youth face both sexual/gender minority stress and cissexism, thereby experiencing compounded minority stress (Hendricks and Testa 2012).

In particular, gender nonconformity is often perceived as a greater threat when it challenges dominant gender norms, particularly for those with male sex assigned at birth. Research in gender socialization suggests that masculinity is more rigidly policed than femininity, and deviations from male gender expectations tend to elicit stronger negative reactions from parents and society (Pascoe 2007; Thorne 1993). Because masculinity is often culturally linked with power and dominance, a transition from a dominant (male) to a subordinate (female or nonbinary) gender position may be viewed by some families as socially devaluing or dishonorable (Connell 1995; Kane 2006). In contrast, gender-atypical expression among those with female sex assigned at birth (e.g., tomboy behaviors) is often more tolerated or even encouraged in early life. These entrenched gender hierarchies and binary norms contribute to disproportionate levels of family rejection among TGD youth with male sex assigned at birth, reinforcing the need for targeted interventions that account for the intersection of gender identity, gender socialization, and familial expectations.

However, these gendered dynamics do not operate in isolation. From an intersectionality perspective (Collins 2019; Crenshaw 1991), experiences of family rejection are also shaped by how gender expression interacts with other axes of identity and structural inequality, including race, ethnicity, religion, and class. For example, cultural and religious beliefs about gender roles may further constrain acceptance of gender nonconformity in some communities, and lower-resourced families may have fewer support systems for navigating SGM identity disclosure (Fortuna et al. 2020; Paceley 2020). Moreover, racialized norms about masculinity and femininity (e.g., expectations about strength, respectability, or obedience) may exacerbate family responses to gender variance in ways that are not captured by gender alone (Battle and Ashley 2008; Schmitz et al. 2020). These interlocking systems of power highlight the need for cultural and contextually sensitive approaches to supporting SGM youth, especially those navigating multiple marginalized identities. These findings suggest that exposure to family rejection is shaped by youths' social location within interlocking systems of power (Collins 2019), even though the present study did not examine intersectional moderation of family rejection effects on outcomes.

Inconsistent with some prior research (Hidalgo et al. 2019; Testa et al. 2015; Toomey et al. 2017), family rejection did not show significant variations for any other sociodemographic variables. This could in part relate to the characteristics of the current sample in which individuals experiencing the highest level of rejection were underrepresented. SGMY in the sample overall experienced low levels of family rejection, although of note, a sizable minority did experience family rejection often. This could reflect the fact that SGMY experiencing higher levels of family rejection were less likely to be able to participate in the current study, such as due to lack of stable housing, lack of consistent Internet/device access, or an inability to find a safe place to participate in program sessions. This finding could also reflect the fact that on average participants were only out to a few family members, limiting their exposure to potential family rejection. It is also possible that SGMY who were most concerned about experiencing family rejection were not out to family members who they believed would engage in rejecting behaviors.

### 4.2 | The Consequences of Family Rejection on Youth Outcomes

Consistent with prior cross-sectional research (Bird et al. 2017; Gamarel et al. 2020; Klein and Golub 2016; Munroe 2018; Ronzón-Tirado et al. 2022; Ryan et al. 2009; Sterzing et al. 2017; Yang et al. 2019), a significant prospective association emerged between family rejection and multiple indicators of psychological and relational problems, including increased depressive symptoms, greater internalized homo/bi/transphobia, and increased TDV perpetration and victimization 5.5 months later. These findings advance the field by demonstrating short-term temporal links between family rejection and both intrapersonal and interpersonal difficulties in a diverse sample of SGMY. Recent work has conceptualized family rejection as a sexual and gender minority-specific adverse childhood experience, highlighting its role as a unique form of identity-based trauma with potential to disrupt healthy development across the life course (Jonas et al. 2022). Importantly, the results offer not only empirical support but also potential refinement to existing frameworks. For example, while PAR theory (Rohner 2004) emphasizes the emotional consequences of perceived withdrawal of parental warmth, our findings suggest that such rejection may also disrupt broader processes of identity integration and relational modeling, domains that extend beyond the original scope of PAR theory.

In addition, youth exposed to rejection from caregivers may be more likely to internalize negative societal attitudes about their identities, thereby reinforcing shame and self-devaluation (Klein and Golub 2016). These feelings may erode relational boundaries and coping resources, increasing the likelihood of engaging in or tolerating aggression in romantic contexts. That family rejection also predicted TDV perpetration suggests that early relational models shaped in the family may carry forward into intimate relationships (Simons and Burt 2011). It is also likely that some negative outcomes of family rejection may increase risk for other negative outcomes, although these relations could not be modeled in the current study. For example, internalized homo/bi/transphobia could increase youth's vulnerability to entering and staying in relationships with high levels of conflict and violence. As another example, depressive symptoms could interfere with youth's ability to regulate their emotions in conflict situations, making it more likely that conflicts escalate to violence. The findings underscore the centrality of family responses in shaping not only SGMY's emotional well-being but also their relational associations during a critical developmental period.

Our findings further underscore the multiple negative impacts of family rejection by demonstrating its prospective associations with diminished hope for the future and lower LGBTQ+ sense of community. Hope has long been recognized as a critical psychological strength associated with positive adjustment, goal pursuit, and mental health (Kwon et al. 2015; Snyder et al. 1991), while a strong sense of community with other LGBTQ+ individuals fosters identity affirmation, social support, and collective resilience (Frost and Meyer 2012; Kertzner et al. 2009). Rooted in positive psychology, hope reflects youth's capacity to envision meaningful goals and develop pathways to achieve them, even in the face of adversity. Family relationships play a central role in fostering hope during adolescence by providing emotional support, reinforcing self-worth, and scaffolding future-oriented thinking (Ackard et al. 2006; Seginer 2008).

From a life course perspective, the adolescence developmental period represents a critical turning point, where identity formation and family responses jointly shape future relational and psychological connections (Meslay et al. 2025). When families engage in rejecting behaviors in response to youths' sexual or gender identity, they not only withdraw emotional support but also undermine the relational foundation through which hope is often cultivated. This disruption may impair youth's ability to set and pursue future goals, leaving them more vulnerable to despair, disengagement, and risk behaviors. Simultaneously, family rejection may discourage youth from seeking connection with affirming LGBTQ+ communities, which are spaces that are crucial for developing identity pride, accessing peer support, and building collective resilience (Frost and Meyer 2012; McConnell et al. 2015).

Consistent with the life course principle of *linked lives*, these findings also highlight how family members' responses to identity disclosure reverberate through adolescents' emotional and social pathways. As such, family rejection not only erodes youth's vision for the future but also constrains their access to vital social resources that could buffer minority stress. Research shows that hope for the future and access to supportive LGBTQ+ communities may serve as protective buffers against minority stress and are associated with lower suicidal ideation among LGBTQ populations (Moe et al. 2023; Hirsch et al. 2012). Yet, as demonstrated in this study, family rejection may erode this critical protective factor, amplifying vulnerability among already marginalized youth. Conversely, there was no evidence of a prospective association between family rejection with some externalizing outcomes (e.g., alcohol use, sexual risk behaviors). It is possible that these behaviors are driven more by peer and social factors (e.g., perceived social norms, peer pressure) than family rejection (Anderson et al. 2011; Lefevor et al. 2024; Windle and Windle 2018). It is also possible that family rejection may relate to these outcomes in the longer term (e.g., increasing risk for the development of problematic substance use in young adulthood), and the salience of family rejection varies across developmental stages, such that family processes may be more strongly linked to internalizing outcomes during adolescence, when family relationships remain highly central, with more distal or indirect effects on externalizing behaviors emerging later in development. An important note is that participants in the current study reported fairly low levels of some of these risk behaviors (e.g., alcohol use), limiting our ability to evaluate association for these outcomes.

### 4.3 | Limitations and Future Directions

There are several study limitations that should be noted. First, the follow-u p period was relatively short, preventing identification of the longer-term impacts of family rejection. We also did not conduct formal mediation or moderation analyses. Given sample size constraints for detecting typically small indirect and interaction effects (Fairchild and MacKinnon 2009), these analyses were beyond the scope of the current study. In addition, because the data were drawn from a larger intervention trial, intervention-related group differences could further complicate interpretation of mediation or moderation pathways. Future research with larger samples and designs specifically powered for mediation and moderation is needed to examine potential mechanisms and buffering processes underlying the associations observed in this study.

Second, the analytic sample consisted of SGMY who were able to participate in an online intervention study and who were out to at least one family member, which likely underrepresents youth experiencing the most severe forms of family rejection (e.g., family expulsion, homelessness, or forced conversion efforts). As such, the findings may not generalize to the most structurally vulnerable SGMY or to those who remain fully concealed within their family systems. The observed associations may represent conservative estimates of the impact of family rejection and should be interpreted as reflecting short-term processes among relatively accessible SGMY rather than population-wide or long-term effects.

Third, we used a shorter and older measure of family rejection. Although newer measures such as LGBTQ Microaggressions and Microaffirmations in Families (Sterzing and Gartner 2020) and the LGBTQ Caregiver Acceptance Scale (Abreu, Gattamorta, et al. 2023) have been created, they are much longer than the measure we used. Given the many measures administered as part of study surveys, we opted to utilize a shorter measure of family rejection. Future research should thus consider methodologies that include more comprehensive measurement (e.g., the full version of the Family Rejection Scale; Ryan et al. 2009) of family rejection and support as well as larger samples of SGMY, longer follow-u p periods, and inclusion of more SGMY experiencing more severe family rejection (e.g., SGMY who have been expelled from the home, SGMY whose caregivers have forced SGMY to undergo conversion therapy).

Fourth, while we created a composite score reflecting the proportion of family members to whom participants were out, we did not collect data on co-residency or frequency of contact with individual family members. This limits our ability to distinguish between disclosure to primary family members versus more peripheral family members, whose impact on youth experiences of rejection may differ. Future studies would benefit from incorporating measures of contact, closeness, and relational centrality to more precisely assess the contextual meaning of outness across family networks.

## 5 | Implications and Conclusion

This study has important implications for prevention and intervention efforts for family social workers and those in related fields. Efforts are needed to reduce family rejection behaviors and increase family accepting behaviors. As an example, the Family Acceptance Project (FAP) is a family-based intervention for caregivers of SGMY that seeks to prevent myriad deleterious outcomes and promote positive youth development for SGMY in the context of their families, cultures, and faith communities. FAP is designed to help families decrease rejection and support and affirm their SGMY (thereby reducing SGMY behavioral, social, emotional, relational, and academic problems; Cohen and Ryan 2021; Ryan 2019a; Ryan 2019b). Pilot research demonstrates the efficacy of FAP in decreasing family rejecting behaviors (Ryan et al. 2018) and reducing post-traumatic stress symptoms among SGMY (Cohen and Ryan 2021; Ryan 2019a). More recently, other promising family interventions have been developed such as Familias con Orgullo/Families with Pride (Lozano et al. 2022), the Parent Support Program (Matsuno and Israel 2021), Parent Resource for Increasing Sexual Minority Support (Goodman and Israel 2020), and Healthy Families Bright Futures (Littleton et al. 2026).

Although this study focused on family-level processes, family rejection occurs within broader structural contexts. Structural inequities such as housing instability and limited access to LGBTQ-affirming schools and mental healthcare may intensify the effects of family rejection and restrict access to protective supports (Hatzenbuehler 2016; Russell and Fish 2016). Macro-level policy efforts remain critical for promoting SGMY well-being. Additionally, future research should also examine how family rejection operates across cultural contexts, where family norms, religious beliefs, and kin networks may shape both its expression and impact differently (Ryan et al. 2010).

In sum, this prospective study adds to the growing body of literature demonstrating that family rejection has damaging effects on the health and well-being of SGMY. Efforts are needed to reduce family rejection and increase family acceptance and support through evidence-based interventions and culturally grounded programs. At the same time, dismantling the broader systems of oppression that perpetuate family rejection and compound minority stress remains a critical goal. Concrete policy actions (e.g., banning conversion therapy, implementing comprehensive nondiscrimination laws in education and healthcare, and preventing school personnel from being compelled to out LGBTQ+ students to unsupportive families) are essential steps toward fostering affirming environments for SGMY. Family social workers and allied professionals play a vital role in both direct service provision and policy advocacy, helping to create safer families, schools, and communities in which all youth can thrive.

## Figures and Tables

**TABLE 1 |:** Sociodemographic characteristics of sample (*N* = 285).

	*n*	%

Condition		
Control	91	31.9
PRYSHM	194	68.1
Sex assigned at birth		
Male	51	17.9
Female	232	81.4
Missing	2	0.7
Gender		
Boy/man	46	16.1
Girl/woman	84	29.5
Transgender	1	0.4
Trans girl/woman	2	0.7
Trans boy/man	38	13.3
Genderqueer	23	8.1
Non-binary	55	19.3
Unsure/questioning	7	2.5
Chose to self-identify	28	9.8
Missing	1	0.4
Racial-ethnic identity		
American Indian or Alaska Native, non-Latino/a/x	3	1.1
Asian, non-Latino/a/x	19	6.7
Black or African American, non-Latino/a/x	44	15.4
White, non-Latino/a/x	126	44.2
Hispanic or Latino/a/x only	12	4.2
Multiracial (includes Hispanic or Latino/a/x)	77	27.0
Missing	4	1.4
Sexual orientation		
Heterosexual/straight	1	0.4
Gay	39	13.7
Lesbian	35	12.3
Pansexual/Bisexual	148	51.9
Queer	32	11.2
Asexual, demisexual, and self-identity	20	7.0
Questioning/Unsure of my sexual orientation	10	3.5
Financial hardship		
No problem at all	124	43.5
A little bit of a problem	115	40.4
A serious problem	30	10.5
A very serious problem	11	3.9
Missing	5	1.8
Relationship Status (past 3 months) (select all)		
Single/not dating	40	14.0
Hooking up but not in a relationship	37	13.0
Friends with benefits	48	16.8
Casual dating relationship	61	21.4
Serious/committed dating relationship	137	48.1
In-person interactions with dating partner (past 3 months)?		
No	57	20.0
Yes	224	78.6
Missing	4	1.4
Region		
Northeast	44	15.4
Midwest	67	23.5
South	104	36.5
West	70	24.6
Ruca		
Metropolitan area core: primary flow within an urbanized area (UA)	238	83.5
Metropolitan area high commuting: primary flow 30% or more to a UA	15	5.3
Micropolitan area core: primary flow within an urban cluster of 10,000 to 49,999 (large UC)	21	7.4
Micropolitan high commuting: primary flow 30% or more to a large UC	3	1.1
Small town core: primary flow within an urban cluster of 2500 to 9999 (small UC)	4	1.4
Small town high commuting: primary flow 30% or more to a small UC	1	0.4
Rural areas: primary flow to a tract outside a UA or UC	3	1.1

**TABLE 2 |:** Descriptive statistics of and correlations among study variables (*N* = 285).

	1	2	3	4	5	6	7	8	9	10	11	12	13	14	15

1. Family rejection (T1)	—	0.12	0.16*	0.12	0.09	0.18**	0.27***	0.17**	0.33***	−0.07	0.05	−0.19*	−0.13	−0.18**	−0.17**
2. TDV perpetration	0.10	—	0.38***	0.23***	0.16*	0.25***	0.05	0.20**	0.06	0.01	0.01	−0.01	0.02	−0.09	−0.03
3. TDV victimization	0.11	0.48***	—	0.40***	0.28***	0.27***	0.05	0.11	0.17**	−0.15*	−0.10	−0.09	−0.16	−0.12	−0.14*
4. Alcohol use	0.05	0.02	0.21**	—	0.48***	0.45***	0.03	0.05	0.13*	−0.06	0.03	0.05	0.04	−0.09	−0.12*
5. Drinking to cope	0.00	0.24***	0.10	0.30***	—	0.40***	0.03	0.07	0.06	−0.11	−0.05	0.04	0.06	−0.06	−0.11
6. Sexual risk behavior	0.15*	0.25***	0.29***	0.30***	0.33***	—	−0.07	−0.02	0.15*	−0.06	0.12*	0.10	0.11	−0.01	−0.04
7. Depressive symptom	0.24**	0.04	0.13	0.07	0.18*	−0.01	—	0.25***	0.09	−0.03	0.03	−0.11	0.02	−0.10	−0.08
8. Impulse control	0.18*	0.01	0.13	0.08	0.11	0.13	0.32***	—	0.17**	−0.01	−0.05	0.09	0.04	−0.11	0.00
9. Internalized HMN	0.32***	0.05	0.02	0.16*	0.13	0.06	0.16*	0.22**	—	−0.20***	−0.19**	−0.08	−0.18*	−0.43***	−0.39***
10. Outness to family SO	−0.03	−0.05	0.06	−0.05	0.01	0.00	0.04	−0.03	−0.13	—	0.55***	0.56***	0.45***	0.11	0.34***
11. Outness to world SO	−0.02	−0.03	0.08	0.02	0.03	0.06	0.06	−0.01	−0.12	0.57***	—	0.36***	0.63***	0.09	0.34***
12. Outness to family GI	−0.04	0.03	0.03	−0.14	0.06	0.15	0.03	0.01	0.01	0.61***	0.39***	—	0.68***	0.06	0.13
13. Outness to world GI	−0.18	0.02	0.02	−0.20*	−0.05	0.12	0.02	−0.08	−0.07	0.36***	0.48***	0.68***	—	0.11	0.28***
14. Hope for the future	−0.27***	−0.02	0.02	−0.10	−0.05	0.02	−0.23**	−0.10	−0.51***	0.16*	0.12	0.00	0.00	—	0.36***
15. Sense of community	−0.20**	−0.04	−0.02	−0.05	0.02	0.02	−0.08	−0.12	−0.44***	0.19**	0.28***	0.10	0.23*	0.56***	—
*M* (T1)	1.13	6.49	13.85	1.11	1.09	2.37	9.97	7.07	1.87	0.70	0.73	0.61	0.70	3.39	3.06
SD (T1)	0.90	22.44	33.91	2.70	1.01	2.01	5.57	2.89	0.60	0.29	0.21	0.38	0.26	0.54	0.71
*M* (T3)	NA	5.32	6.12	1.34	1.02	2.02	9.29	6.68	1.86	0.71	0.74	0.61	0.69	3.33	3.00
SD (T1)	NA	34.35	17.98	3.33	0.96	2.15	6.08	3.00	0.63	0.30	0.22	0.38	0.28	0.58	0.84

*Note:* Correlations among study variables measured at T1 are shown in the higher triangle. Correlations among study variables measured at T3 are shown in the lower triangle. Abbreviations: GI = gender identity; HMN = homo/bi/transphobia; *M* = mean; SD = standard deviation; SO = sexual orientation; TDV = teen dating violence.

* *p* < 0.05.

** *p* < 0.01.

*** *p* < 0.001.

**TABLE 3 |:** Tests of variation in family rejection by demographic groups (Research Question 1).

ANOVA	Family rejection						
	*n*	*M*	SD	*F*	df	*η* ^2^	*p*

Age				1.25	(3, 250)	0.02	0.291
15 years old	37	1.27	0.85				
16 years old	69	1.24	0.95				
17 years old	118	1.06	0.88				
18 years old	30	0.96	0.94				
Gender identity				2.47	(4, 249)	0.04	0.045
AMAB cis	34	0.80	0.96				
AFAB cis	64	1.11	0.83				
AMAB TGD	15	1.60	0.85				
AFAB TGD	138	1.17	0.91				
Intersex/Missing	3	0.67	0.52				
Racial-ethnic identity				1.06	(5, 244)	0.02	0.383
American Indian/Alaska Native	3	1.58	1.38				
Asian	14	0.88	0.59				
Black or African American	34	0.86	0.77				
White	117	1.18	0.92				
Hispanic or Latine Only	10	1.17	0.96				
Multiracial	72	1.13	0.92				

*t*-test		*M*	SD	*t*	df	*d*	*p*

Sexual orientation				0.71	252	0.10	0.476
Monosexual	69	1.06	0.98				
Non-monosexual	185	1.15	0.88				

Abbreviations: AFAB cis = assigned female youth who identified as cisgender; AFAB TGD = assigned female youth who identified as trans and gender diverse; AMAB cis = assigned male youth who identified as cisgender; AMAB TGD = assigned male youth who identified as trans and gender diverse.

**TABLE 4 |:** Results of path analysis relating T1 family rejection to T3 deleterious and resilience promoting factors (Research Question 2).

DV	*b*	SE	*p*	*β*	*R*^2^/IRR^a^

Model 1					
TDV perpetration	**0.29**	**0.05**	**< 0.001**	**0.29**	**1.34**
TDV victimization	**0.36**	**0.03**	**< 0.001**	**0.35**	**1.42**
Model 2					
Alcohol use	−0.01	0.22	0.966	−0.003	0.33
Drinking to cope	−0.05	0.07	0.483	−0.05	0.29
Sexual risk behavior	0.13	0.15	0.396	0.06	0.32
Model 3					
Depressive symptoms	**1.02**	**0.48**	**0.032**	**0.15**	**0.21**
Impulse control difficulties	0.16	0.21	0.450	0.05	0.35
Model 4					
Internalized homo/bi/transphobia	**0.09**	**0.05**	**0.046**	**0.13**	**0.37**
Outness to family – sexual orientation	−0.01	0.02	0.708	−0.02	0.63
Outness to world – sexual orientation	−0.02	0.01	0.145	−0.08	0.52
Outness to family – gender identity	0.01	0.03	0.722	0.02	0.57
Outness to world – gender identity	−0.04	0.02	0.064	−0.12	0.57
Hope for the future as an LGBTQ person	**−0.09**	**0.04**	**0.029**	**−0.14**	**0.31**
Sense of LGBTQ+ community	**−0.12**	**0.05**	**0.027**	**−0.13**	**0.37**

*Note:* Bold represents significance *p* < 0.05. T1 = Time 1 (baseline). T3 = Time 3 (5.5 months after baseline). DV's were grouped in models by construct (Model 1: TDV; Model 2: risk behavior; Model 3: psychological/emotional problems; Model 4: LGBTQ+ specific risk/resilience). Model 1: H0 = −6164.37, AIC = 12362.73, BIC = 12424.83, SABIC = 12370.92. Model 2: *χ*^2^(10) = 21.26, *p* = 0.019, CFI = 0.95, TLI = 0.92, RMSEA = 0.06, SRMR = 0.05. Model 3: *χ*^2^(5) = 7.06, *p* = 0.217, CFI = 0.99, TLI = 0.98, RMSEA = 0.04, SRMR = 0.03. Model 4: *χ*^2^(50) = 79.24, *p* = 0.005, CFI = 0.97, TLI = 0.96, RMSEA = 0.05, SRMR = 0.06.

Abbreviation: TDV = teen dating violence.

a Incidence rate ratio (IRR) and *R*^2^ were used for effect sizes. IRR was used for count DVs (Model 1). *R*^2^ was used for continuous DVs (Models 2–4). For IRR, closer to 1 represents smaller effects. For *R*^2^, small = 0.02, medium = 0.13; large = 0.26; Cohen 1992.

## Data Availability

The data that support the findings of this study are available from the corresponding author upon reasonable request.
